# The Role of the Cystic Fibrosis Transmembrane Conductance Regulator (CFTR) in Enamel Formation

**DOI:** 10.1007/s00223-026-01539-1

**Published:** 2026-05-04

**Authors:** Jake Ngu, Charles M. Met, Atoosa Heidari Bigvand, Yan Zhang, Pamela K. Den Besten

**Affiliations:** 1 Department of Orofacial Sciences, School of Dentistry, University of California, San Francisco, San Francisco, USA; 2 Oral and Craniofacial Sciences Graduate Program, School of Dentistry, University of California, San Francisco, San Francisco, USA; 3 School of Dentistry, University of California, San Francisco, San Francisco, USA; 4 Center for Oral Health Research, University of California, San Francisco, San Francisco, USA

**Keywords:** Ameloblasts, CFTR, Cystic fibrosis, Enamel, pH, Modulation

## Abstract

Mutations in the cystic fibrosis transmembrane conductance regulator (CFTR) cause cystic fibrosis (CF), an autosomal recessive multiorgan disorder. In addition to respiratory, digestive, and reproductive pathologies, patients with CF also exhibit higher rates of enamel defects. CFTR is an anion channel expressed in maturation-stage ameloblasts and is known for its role in chloride and bicarbonate conductance, a function thought to promote enamel mineralization through the neutralization of protons released during hydroxyapatite formation. Previous studies of CFTR global knockout mice have shown hypomineralization and acidification of the maturation-stage enamel matrix. However, the systemic nature of CF and the contribution of extrinsic factors may contribute to these findings. To determine how CFTR specifically contributes to amelogenesis, we generated a conditional knockout mouse in which CFTR loss-of-function is driven by Cre expression under the promoter of the maturation-stage ameloblast marker, *Odam.* CFTR loss-of-function resulted in the formation of hypomineralized enamel. The enamel matrix was acidified, and loss of matrix pH cycling coincided with arrested ameloblast modulation as indicated by failed re-localization of tight/ gap junctions and ion transporters to the ameloblast distal membrane. Amelogenin hydrolysis was reduced and retained in the maturing enamel matrix, which was associated with reduced *Klk4* expression and activity. Transcriptional analyses of incisor ameloblasts showed reduced expression of acid-base regulators, consistent with dysregulation of both intracellular and extracellular pH. Together, these findings show multiple functions of CFTR in incisor enamel maturation, including regulation of intra/ extracellular pH, ameloblast modulation, and extracellular matrix remodeling.

**Supplementary Information:**

The online version contains supplementary material available at 10.1007/s00223-026-01539-1.

## Introduction

The cystic fibrosis transmembrane conductance regulator (CFTR) is an ATP and phosphorylation-dependent ion channel that conducts anions, most notably chloride and bicarbonate, across cell membranes to regulate salt, water, and pH homeostasis [1–3]. Mutations in the human *CFTR* gene cause cystic fibrosis (CF), a genetic condition that follows an autosomal recessive pattern of inheritance [4, 5]. While most known for its debilitating effects on the airway epithelium, where failure to conduct chloride and bicarbonate into the respiratory tract leads to thickening of secretions and obstruction of the airway, CF is also a multisystem disorder that affects numerous organs including the pancreas, intestines, sweat glands, salivary glands, uterus, vas deferens, and bone [6]. Patients with CF also present with higher rates of tooth enamel defects compared to individuals without CF [7].

Wright et al. first describe the enamel phenotype in CFTR global knockout mice [8]. Bronckers et al. immunolocalized CFTR to the apical membrane of maturation-stage ameloblasts [9] and showed that CFTR loss-of-function resulted in an entirely acidic enamel matrix [10]. This is contrary to normal developing enamel, which contains cyclically alternating bands of neutral (~ pH 7.2) and acidic (~ pH 5.4–6.5) enamel matrix, known as pH cycling [11, 12]. These zones are associated with maturation-stage ameloblasts of two distinct morphologies [13]. The longer acidic bands of enamel matrix are overlain with the so-called ruffle-ended ameloblasts, which are enriched with numerous ion channels, exchangers and transporters [14–17], and contain numerous apical membrane invaginations that mediate the endocytosis of enamel matrix proteins. In contrast, zones of neutral pH are overlain with smooth-ended ameloblasts, which lack membrane invaginations [17, 18]. Many of the membrane proteins associated with the ruffle-ended ameloblast apical border are noticeably absent from the apical membrane of smooth-ended cells and are trafficked/ re-localized to the cytosolic space [14–16].

Enamel matrix pH cycling is thought to be regulated by the coordinated movement of chloride and bicarbonate into the forming enamel, as a means of neutralizing protons released during hydroxyapatite formation [19–21]. This is supported by the identification of numerous transporters and exchangers that facilitate the transepithelial movement of chloride and bicarbonate in maturation-stage ameloblasts. These include the electrogenic sodium/ bicarbonate cotransporter 1 (NBCe1), anion exchanger 2 (AE2), and members of the SLC26 family of chloride/ bicarbonate exchangers [22–24]. Additionally, carbonic anhydrases (CA), namely CA2 and CA6, have also been shown to be expressed in ameloblasts and are thought to also contribute to bicarbonate-mediated pH regulation [20, 25]. Mutations in many of these bicarbonate-related genes have been shown to cause enamel defects in both humans and animal models, highlighting the importance of pH regulation in enamel formation [23, 26–28].

To investigate the relationship between pH cycling and ameloblast modulation, we generated a maturation-stage ameloblast-specific CFTR conditional knockout mouse by utilizing the *Odam-IRESCre* mouse line, which drives Cre expression under the *Odam* promoter. ODAM is highly upregulated in maturation ameloblasts, though its loss-of-function does not affect enamel formation [29]. Therefore, the effects of inserting the IRES-Cre cassette downstream of the *Odam* promoter on *Odam* expression would have minimal contributions to assessing the effect of CFTR loss-of-function on enamel formation. The generation of *Odam-Cre*,* Cftr* ^*fl/fl*^ mice conditionally ablated CFTR from the maturation stage, allowing us to ascertain the primary role of CFTR in directing pH cycling, ameloblast modulation, and enamel matrix maturation [30].

## Methods

### Animals

Wild-type (*wt)* mice (C57BL/6J | RRID: IMSR_JAX:000664) and *Odam-IRESCre* mice (C57BL/6J-*Odam*^*em1(cre)Mlp*^/Mmjax | RRID: MMRRC_067402-JAX) were obtained from Jackson Laboratory. *Cftr* ^*fl/fl*^ mice (*Cftr*^*tm1Cwr*^) were generously provided by Dr. Michelle Yu, MD, PhD at UCSF. It should be noted that the original publication that described this *Cftr* ^*fl/fl*^ mouse model refers to the mice as having loxP sites flanking *Cftr* exon 10 [30]. However, this exon has since been renumbered to exon 11 and will be the nomenclature used moving forward to avoid confusion (refer to NCBI Reference Sequence: NM_021050.2). Exon 11 encodes for a portion of the CFTR NBD1 domain, which controls channel gating. Excision of this exon produces an in-frame mRNA transcript but non-functional CFTR protein [30].

Experimental mice were obtained by crossing *Odam-IRESCre* mice (referred to as *Odam-Cre*) with *Cftr* ^*fl/fl*^ mice. Genotyping was performed by polymerase chain reaction (PCR) utilizing crude DNA extracts derived from tail snips lysed with alkaline lysis buffer (40 mM NaOH, 0.2 mM EDTA) and neutralized with 40 mM Tris-HCl (pH 5). PCR was performed utilizing Taq DNA Polymerase Kit (Invitrogen™, 10342020). Products were run on 1.5% agarose gel with GelRed™ Nucleic Acid Gel Stain (Biotium, 41003) and visualized by UV transilluminator. Refer to Supplemental Table 1 for genotyping primers, which are from Jackson Laboratory for the *Odam-Cre* allele or Hodges et al. [30] for the *Cftr* ^*fl/fl*^ allele. *Odam-Cre* mice were used as controls to account for Cre toxicity, off-target cutting, and impact of Cre insertion into the endogenous *Odam* locus on expression of *Odam* (Supplemental Fig. 1) [31]. All animals were maintained in the UCSF animal care facility, a barrier facility accredited by the Association for Assessment and Accreditation of Laboratory Animal Care (AAALAC). Experimental procedures in this study were approved by the Institutional Animal Care and Use Committee (IACUC) under the protocol AN200166-00 F.

### Histology and Immunohistochemistry

Postnatal 6-week-old (P6W) *wt*,* Odam-Cre*,* Cftr* ^*fl/fl*^, and *Odam-Cre; Cftr* ^*fl/fl*^ mice were euthanized by CO_2_ inhalation, followed by cervical dislocation. Hemimandibles were dissected free of surrounding soft tissue and fixed in 4% paraformaldehyde (PFA) overnight at 4 °C. Samples were then decalcified in 8% EDTA at 4 °C for 2 weeks, with daily EDTA changes. The hemimandibles were then processed, paraffin-embedded, and sectioned along the sagittal plane to a thickness of 5 μm. Sections were then deparaffinized, rehydrated, and either stained with hematoxylin and eosin (H&E) or subjected to antigen retrieval for immunohistochemistry (IHC). Following antigen retrieval, sections were permeabilized with 0.1% Triton X-100 and endogenous peroxidase activity was blocked with 3% H_2_O_2_. Sections were then blocked with Trident Universal Protein Blocking Reagent (GeneTex, Inc., GTX30963) for 1 h, followed by overnight incubation at 4 °C with primary antibodies against CFTR, ODAM, ATP6V1A, ACTB, CLDN1, GJA1, NCKX4, AMELX, CLTC, LAMP1, and EEA1 (see Supplemental Table 2 for dilution factors). Following overnight incubation, sections were washed with 0.04% phosphate buffered saline tween (PBS-T) and incubated with anti-rabbit IgG HRP Conjugate (Promega W401) or Goat anti-Rabbit IgG (H + L) Secondary Antibody, FITC (Thermo Fisher Scientific Inc., 31635). ImmPACT^®^ DAB Substrate Kit, Peroxidase (Vector^®^ SK-4105) was used to visualize HRP labeled sections, which were then counterstained with hematoxylin. For fluorescently labelled slides, sections were counterstained with Hoechst 33,342 (Life Technologies). Slides were viewed under a Nikon Eclipse E800 microscope and images were taken with an Olympus DP74-CU camera.

### RNA Extraction, RT-PCR, and RT-qPCR

Characterization of *Cftr* expression was performed by reverse transcription quantitative PCR (RT-qPCR) of lower first molar enamel organs microdissected free of underlying pulp from *wt* mice at postnatal day 5 (P5), P7, P8, P9, P11, and P13. A total of three pups were pooled for each biological replicate (*N* = 3 replicates) to ensure sufficient RNA yield. For RT-PCR validation of successful floxing of the *Cftr* exon 11 in *Odam-Cre; Cftr* ^*fl/fl*^ mice, hemimandibles were divided along the distal end of the second molar to separate secretory from the maturation stage, as previously described [24]. Maturation-stage enamel organs from two mice were pooled together to ensure sufficient yield. A total of *N* = 4 biological replicates were used for RT-qPCR. Samples were lysed in TRIzol™ Reagent (Ambion^®^, 15596018) by agitation and passing through a 23-gauge needle. Total RNA was purified by Direct-zol™ RNA MiniPrep kit (Zymo Research, R2052). RNA concentration was quantified with Thermo Scientific™ NanoDrop™ One Spectrophotometer and cDNA libraries were generated using SuperScript™ III Reverse Transcriptase (Invitrogen™, 18080093) with 250 ng of total RNA as input. RT-PCR was performed using cDNA libraries as template and as described for genotyping. RT-qPCR was performed using the iTaq™ Universal SYBR^®^ Green Supermix (Bio-Rad Laboratories, Inc., 1725120) in a QuantStudio™ 6 Pro Real-Time PCR System. *Actb* was used as a housekeeping gene and the ΔΔCt method was used to calculate fold change of *Odam-Cre; Cftr* ^*fl/fl*^ relative to controls. ΔCt values were used to calculate statistical significance by the independent Student’s t-test or the multiple t-test with Bonferroni correction following one-way ANOVA. *P <* 0.05 was significant or adjusted for multiple comparison correction. See Supplemental Table 1 for PCR primers.

### Micro Computed Tomography (µCT)

Hemimandibles from P6W *Odam-Cre*, and *Odam-Cre; Cftr* ^*fl/fl*^ mice were collected as described above and fixed in 4% PFA overnight at 4 °C. Samples were transferred to PBS and shipped to Micro Photonics in Allentown, PA for scanning on a SkyScan 1275 microCT system. Briefly, scans were performed at 10 W of power with no filter at 40 kV/250uA of voltage with an isotropic voxel size of 8 μm. Once complete, scans were uploaded into Amira software (ThermoFisher). A non-local means filter [spatial StdDev = 5, intensity StdDev = 0.2, search window [px] = 9, local neighborhood [px] = 3] and an unsharp masking [interpretation = 3D, edge size [px] = 6, edge contrast = 0.5, brightness threshold = 0] image processing filter was applied to increase the contrast between the enamel and the surrounding mineralized tissue. Representative 2D images of the scanned samples were taken along the sagittal and coronal planes, with the coronal sections being taken at the gingival margin of the incisor and the distal/ mesial ends of the first molar.

### Enamel Matrix pH Staining

The full length of hemimandible incisors were microdissected and liberated from the surrounding alveolar bone. Incisors were then cleaned with a moist, ice-cold Kimwipe to remove the overlaying enamel organ and immediately immersed in a methyl red solution (0.2% methyl red in 95% methanol). Incisors were stained for 45 s to 1 min and were subsequently air-dried. Images of the incisors were taken on a Leica dissecting microscope.

### Enamel Matrix Protein Extraction and Assessment by SDS-PAGE/ Western Blot

Maturation-stage incisor fragments were microdissected free of the surrounding alveolar bone of P6W *Odam-Cre* and *Odam-Cre; Cftr* ^*fl/fl*^ hemimandibles. These incisor fragments were cleaned free of the overlaying enamel organ and incubated in 0.17 N HCl/0.95% formic acid with gentle shaking for 2 h at 4 °C, with vortexing at max speed every half-hour for 30 s. After incubation, samples were centrifuged to pellet undissolved solids, and the crude extract was subjected to buffer exchange with 0.01% formic acid using a centrifugal 3 K-filter unit (Amicon^®^ UFC5003) [32]. Equal volumes of control and conditional knockout enamel matrix extracts were mixed with 2X SDS loading buffer. Samples were loaded onto 4%–20% Mini-PROTEAN TGX SDS-PAGE gels (Bio-Rad, 4561094) and subjected to electrophoresis with Tris/glycine/SDS running buffer at 100 V for 90 min. Gels were then either stained with Coomassie Brilliant Blue or transferred to PVDF membranes (Invitrogen™ IB401001) using an Invitrogen iBlot™ Gel Transfer Device. Membranes were blocked with Intercept Protein Free Blocking Buffer (LI-COR, 927-90001) and then incubated overnight with either anti-AMELX or anti-KLK4 antibodies (see Supplemental Table 2 for dilutions). Blots were then washed with 0.1% PBS-T and incubated with IRDYE 680RD donkey anti-rabbit IgG secondary antibody (LICORbio™, 926-68073) and imaged on the Odyssey XF imager.

### KLK4 Matrix Activity

*N* = 4 biological replicates of microdissected maturation-stage incisor fragment from P6W *Odam-Cre* and *Odam-Cre; Cftr* ^*fl/fl*^ hemimandibles were directly incubated with a fluorogenic quench peptide substrate with a KLK4 specific cleavage site, Boc-V-P-R-AMC, (R&D Systems, ES011) in KLK4 activity buffer (50 mM Tris-HCl, 150 mM NaCl and 10 mM CaCl_2_) [33]. Fluorescent readout was measured using a SpectraMax iD3 fluorescence plate reader (Molecular Devices) at excitation of 380 nm and emission at 460 nm. Measurements were taken every 2.5 min over 100 min. Statistical significance comparing control and conditional knockout samples across time points were calculated using the two-tailed, unpaired t-test in GraphPad Prism software with *p* < 0.05 as significant.

### In Situ Hybridization RNAScope

P6W *Odam-Cre* and *Odam-Cre; Cftr* ^*fl/fl*^ sections were subjected to in situ hybridization RNAScope utilizing the RNAscope^®^ 2.5 HD Detection Reagents-RED kit (Advanced Cell Diagnostics Inc., 322360). Sections were boiled in target retrieval buffer for 15 min and then pre-treated with protease plus solution at 40 °C for 15 min. The sections were incubated with *Klk4* probes for 2 h. After a series of amplification steps, sections were incubated with Fast Red complex to visualize *Klk4* expression. All incubation steps were performed in the HybEZ™ II Hybridization Oven. Sections were then counterstained with hematoxylin. Slides were imaged as described above.

## Results

### CFTR Transcripts Reflect the Deletion of Exon 11 in Odam-Cre; Cftr fl/fl Enamel Organs

**Fig. 1:**
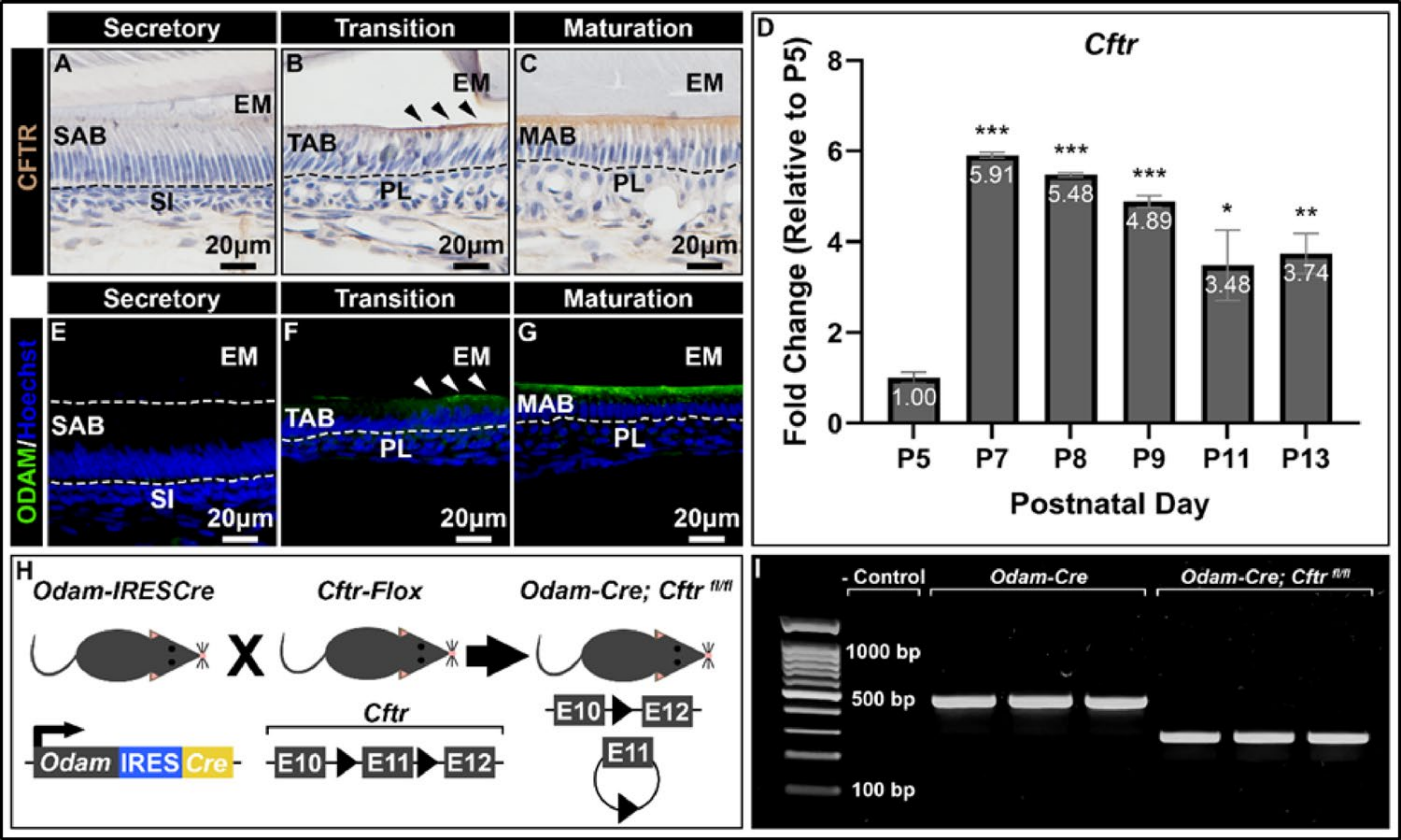
**Generation of a maturation-stage ameloblast-specific CFTR conditional knockout mouse.** 40x representative images of *wt* hemimandible sagittal sections immunostained with anti-CFTR monoclonal antibody at (**A)** secretory, (**B)** transition, and (**C)** maturation stages reveal CFTR to localize specifically to the apical membrane of incisor maturation-stage ameloblasts. Immunoreaction is in brown, and nuclei are in blue. SAB, secretory-stage ameloblasts; TAB, transition-stage ameloblasts; MAB, maturation-stage ameloblasts; SI, stratum intermedium; EM, enamel matrix; PL, papillary layer. (**D)** Reverse transcription quantitative polymerase chain reaction (RT-qPCR) of *Cftr* expression in first molar enamel organs at different developmental timepoints demonstrates that transition-stage (P7) and maturation-stage enamel organs (P8-P13) have higher *Cftr* expression relative to secretory-stage enamel organs (P5). Fold change was calculated relative to P5 by the ΔΔCT method, followed by two-tailed, unpaired t-test with Bonferroni correction following ANOVA (α = 0.01). * *p* < 0.01; ** *p* < 0.005; *** *p* < 0.00005. 40x representative images of *wt* hemimandible sagittal sections immunostained with anti-ODAM polyclonal antibody reveals ODAM is immunonegative at the (**E)** secretory stage but stains positively at the **(F-G)** onset of enamel maturation (see arrows) and during the maturation stage, specifically at the distal end of maturation-stage ameloblasts. Immunoreaction is in green and nuclei counterstaining is in blue. (**H)** Schematic diagram of the breeding strategy used to generate the maturation-stage specific CFTR conditional knockout mouse, where Cre expression is driven by the *Odam* promoter. Cre expression in *Odam*-expressing cells leads to *Cftr* exon 11 excision and production of non-functional CFTR. (**I)** Gel electrophoresis of reverse transcription polymerase chain reaction (RT-PCR) products generated from maturation-stage enamel organs utilizing primers flanking *Cftr* exon 11 demonstrates successful floxing of exon 11 (192 bps) in *Odam-Cre; Cftr* ^*fl/fl*^ mice whereas *Odam-Cre* controls retain the full-length amplicon

In *wt* mouse incisors, CFTR protein expression began at the onset of enamel maturation, where it immunolocalized to the apical, enamel-facing edge of ameloblasts (Fig. 1A-C). Similarly, in molars, *Cftr* gene expression was dramatically increased during the transition stage (P7) and continued to be upregulated through the maturation stage (P8-P13), relative to the secretory stage (P5) (Fig. 1D). The spatiotemporal expression pattern of CFTR coincided with that of ODAM, as both immunolocalized to the apical edge of maturation-stage ameloblasts and began to be synthesized at the transition stage (Fig. 1E-G) and throughout the maturation stage (Supplemental Fig. 2).

*Odam* expression is significantly reduced in the *Odam-Cre* mice (Supplemental Fig. 1); however, we did not find evidence of morphological differences between the *Odam*^*+/+*^, *Odam*^*Cre/+*^, and *Odam*^*Cre/Cre*^ mouse models (data not shown), which is expected as ODAM loss-of-function mice do not have changes in enamel formation [29]. Furthermore, we used the *Odam-Cre* mice as controls for all studies of *Cftr* loss of function in the *Odam-Cre*,* Cftr* ^*fl/fl*^ mice; therefore, any changes in enamel formation can be ascribed to the absence of CFTR.

In crossing the *Odam-Cre* mouse line with the *Cftr* ^*fl/fl*^ mouse to specifically ablate CFTR from maturation-stage ameloblasts (Fig. 1H), the critical 192 bp exon 11 is excised from *Cftr* to result in a maturation-stage ameloblast-specific conditional knockout mouse (Fig. 1I). Exon 11 encodes for a portion of the NBD1 domain, which controls channel gating. Loss of this exon leads to an in frame mRNA transcript but non-functional CFTR protein, which can still be detected by immunostaining in *Odam-Cre; Cftr* ^*fl/fl*^ mice (Supplemental Fig. 3) [30].

### Targeted Loss of CFTR From Maturation-Stage Ameloblasts Results in Acidified and Hypomineralized Enamel

**Fig. 2:**
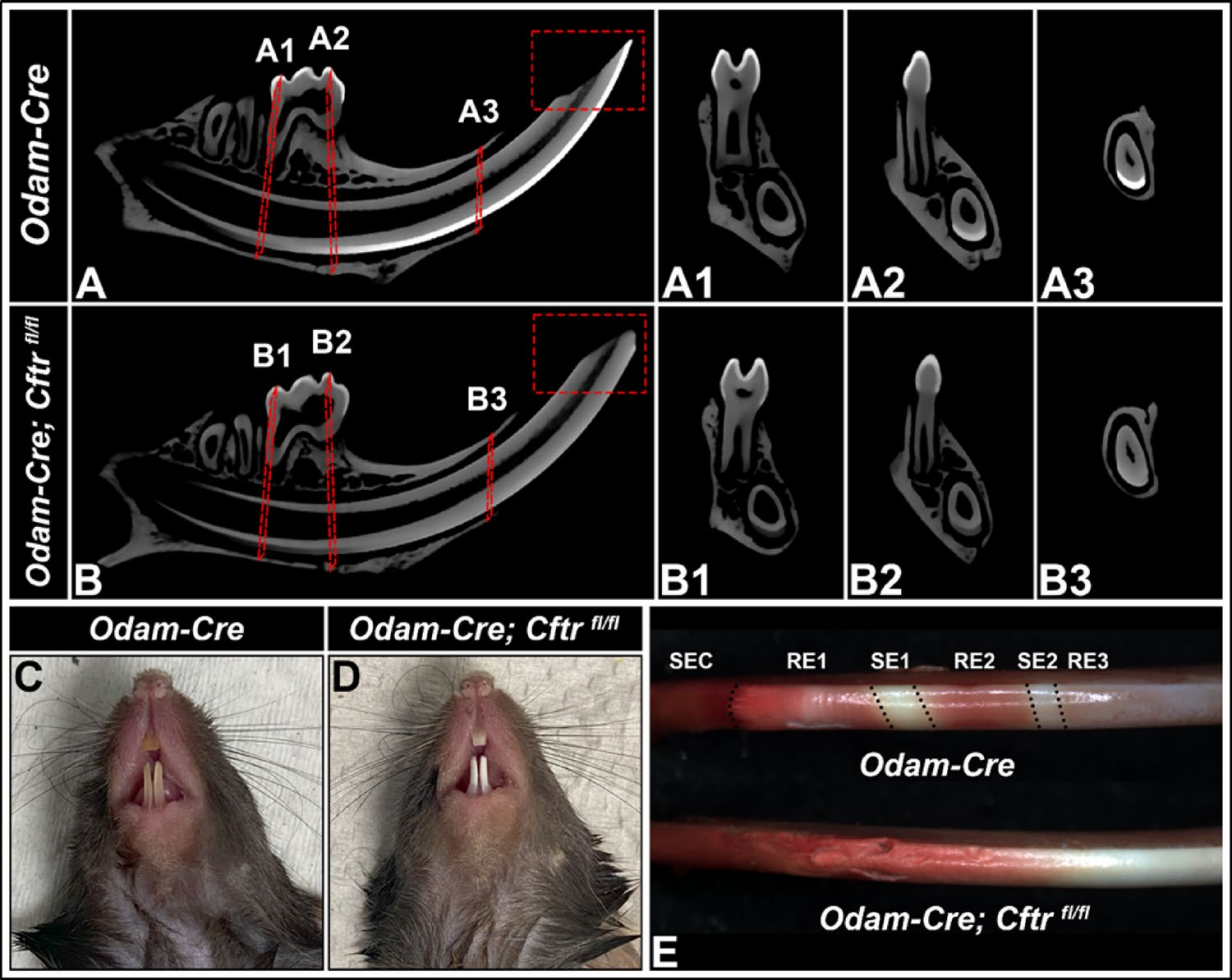
**Characterization of enamel defects in CFTR conditional knockout mice.** Representative 2D micro-computed tomography (µCT) sagittal sections of (**A)** *Odam-Cre* and (**B)** *Odam-Cre; Cftr* ^*fl/fl*^ mice along with coronal sections taken at (**A1**, **B1**) the distal end of the 1st molar, (**A2**, **B2**) the mesial end of the 1st molar, and (**A3**, **B3**) the gingival margin of the incisor (red box) showing increased wear along the incisal edge of the CFTR conditional knockout mice, show that the incisor enamel is hypomineralized. Representative images of incisors from (**C)** *Odam-Cre* and (**D)** *Odam-Cre; Cftr* ^*fl/fl*^ mice show that conditional knockout mouse incisors have chalkier, whiter enamel relative to controls. Incisors also appear worn down and fractured along the dentinoenamel junction.** (E)** Staining of microdissected mouse mandibular incisors with methyl red pH indicator reveals that conditional deletion of CFTR from maturation-stage ameloblasts leads to defective pH cycling. Conditional knockout incisors remain acidic (red) whereas control incisors cyclically modulate between bands of acidic pH and bands of neutral pH (yellow). SEC, secretory; RE, ruffle-ended; SE, smooth-ended

µCT scans of *Odam-Cre* control and *Odam-Cre; Cftr* ^*fl/fl*^ hemimandibles revealed that functional ablation of CFTR from maturation-stage ameloblasts resulted in the formation of hypomineralized enamel. Relative image intensity of both incisors and molars was reduced in conditional knockout mice hemimandibles, though the phenotype appeared much more severe in incisors, relative to molars (Fig. 2A-B). Knockout incisor tips were noticeably blunted compared to controls (see red boxes), suggesting a higher susceptibility to wear. Relative image intensity of knockout incisor enamel at early maturation (Fig. 2A1, B1), mid maturation (Fig. 2A2, B2), and near eruption (Fig. 2A3, B3) were all reduced relative to control enamel, and the radiopacity of the enamel was near indistinguishable from the overlaying dentin at all stages of enamel maturation. This corresponds with gross assessment of *Odam-Cre* and *Odam-Cre; Cftr* ^*fl/fl*^ incisors, where knockout incisors appeared chalky, white, and more noticeably worn down at the tip (Fig. 2C-D). Staining with methyl red pH indicator showed a loss of pH cycling (Fig. 2E) in conditional knockout incisors as they remained acidic and failed to cycle between bands of acidic and neutral pH. This cycling is apparent in the *Odam-Cre* control incisors, where acidic bands stain red-orange and neutral bands stain more yellowish white.

### CFTR Loss-of-Function Disrupts Maturation-Stage Ameloblast Cell Morphology and Polarity

**Fig. 3:**
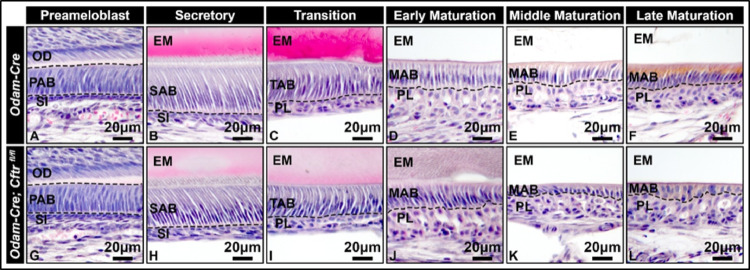
**Histological analyses of different enamel formative stages in CFTR conditional knockout mice.** 40x representative images of *Odam-Cre* and *Odam-Cre; Cftr* ^*fl/fl*^ hemimandibles sectioned along the sagittal plane and stained with hematoxylin and eosin (H&E) reveal no obvious cell morphological differences at the (**A**, **G**) preameloblast, (**B**, **H**) secretory, or (**C**, **I**) transition stages of amelogenesis. During (**D**, **J**) the early maturation stage, *Odam-Cre; Cftr* ^*fl/fl*^ ameloblasts exhibit a more disorganized epithelial cell layer relative to *Odam-Cre* control ameloblasts. At (**E**, **K**) mid-maturation and into the (**F**, **L**) later stages of enamel maturation, CFTR conditional knockout ameloblasts continue to exhibit a disorganized epithelial cell layer but also appear to be shorter relative to controls, demonstrating signs of either premature degeneration or loss of epithelial cell polarity. PAB, preameloblasts; SAB, secretory-stage ameloblasts; TAB, transition-stage ameloblasts; MAB, maturation-stage ameloblasts; OD, odontoblasts; SI, stratum intermedium; PL, papillary layer

H&E-stained sagittal sections of *Odam-Cre* control and *Odam-Cre; Cftr* ^*fl/fl*^ hemimandibles showed no morphological differences during the preameloblast (Fig. 3A, G) and secretory stages (Fig. 3B, H). During the transition stage (Fig. 3C, I), where CFTR begins to be expressed, there was no obvious morphological change either. However, early maturation-stage ameloblasts exhibited a slightly more disorganized cell layer (Fig. 3D, J). As enamel maturation progresses, this phenotype became more severe (Fig. 3E, K). In the later stages (Fig. 3F, L), maturation-stage ameloblasts appeared to lose organization of their characteristic single-layer epithelial sheet. Qualitatively, these cells appeared shorter compared to controls and seemed to lose their epithelial polarity. *Odam-Cre* control ameloblasts did not exhibit obvious morphological differences compared to C57BL/6 *wt* and *Cftr* ^*fl/fl*^ ameloblasts (Supplemental Fig. 4).

### Ameloblasts Do Not Modulate When CFTR is Conditionally Knocked Out From Maturation-Stage Ameloblasts

**Fig. 4:**
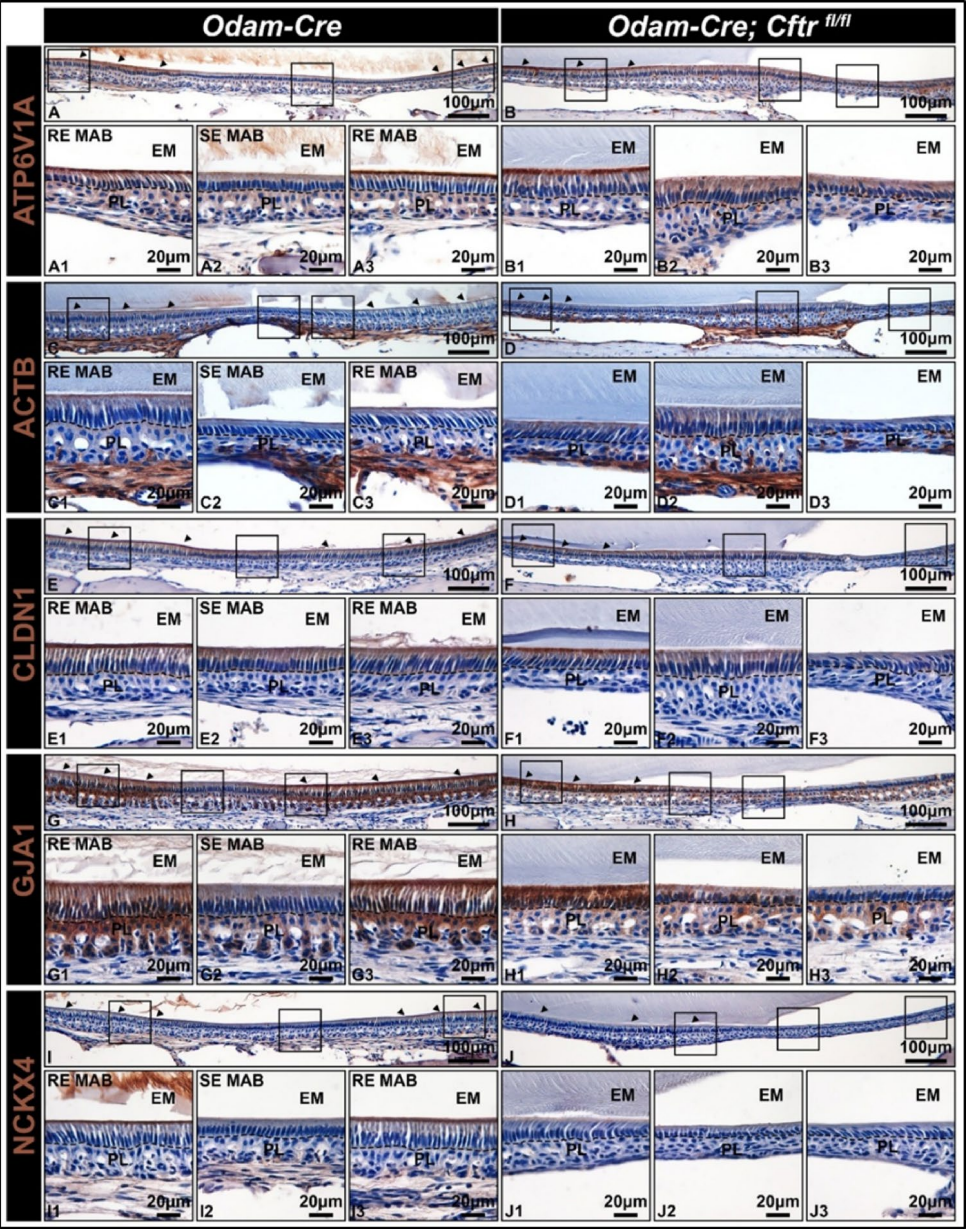
**Assessment of ameloblast modulation in CFTR conditional knockout mice.** 10x and 40x representative images of hemimandible sagittal sections immunostained for (**A-B**) ATP6V1A, (**C-D**) ACTB, (**E-F**) CLDN1, (**G-H**) GJA1/ Cx43, and (**I-J**) NCKX4 demonstrate that *Odam-Cre* controls cycle between ruffle-ended (RE MAB) and smooth-ended (SE MAB) ameloblasts; however, *Odam-Cre; Cftr* ^*fl/fl*^ form an initial ruffled border that transitions to a SE MAB-like phenotype, which fails to cycle back to RE MABs. Immunoreaction is in brown and counterstaining of nuclei is in blue. PL, papillary layer; EM, enamel matrix

Since loss of CFTR led to an acidified matrix and altered ameloblast morphology, we next wanted to more precisely examine CFTR’s role in regulating ameloblast modulation. To do this, we immunostained for proteins that cyclically re-localize from the apical border of ruffle-ended cells to a more intracellular localization in smooth-ended cells. This included V-ATPase subunit ATP6V1A (Fig. 4A-B), beta actin (ACTB) (Fig. 4C-D), the tight junction protein claudin 1 (CLDN1) (Fig. 4E-F), the gap junction alpha protein 1/ connexin 43 (GJA1/ Cx43) (Fig. 4G-H), and the potassium-dependent, sodium/ calcium exchanger 4 (NCKX4) (Fig. 4I-J). We found that CFTR-deficient MABs formed an initial ruffled border, where all these markers localized to the distal membrane. However, these cells then appeared to adopt a smooth-ended-like morphology that never reformed a ruffled border, whereas control MABs continued to cyclically re-localize these proteins to and from the apical membrane. See Supplemental Fig. 5 for staining negative controls.

### pH Regulators Are Transcriptionally Downregulated In Incisor Enamel Organs With CFTR Loss-of-Function

**Fig. 5:**
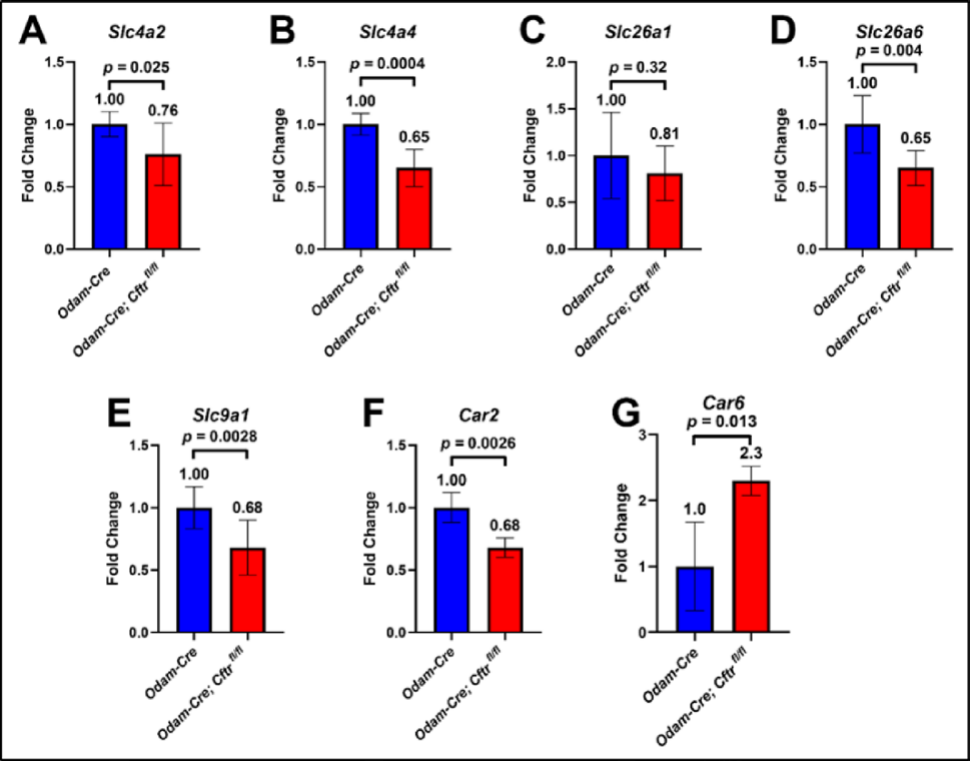
**Expression analysis of pH regulators in CFTR conditional knockout incisor enamel organs.** Reverse transcription quantitative polymerase reaction (RT-qPCR) of microdissected incisor maturation-stage enamel organs from *Odam-Cre* and *Odam-Cre; Cftr* ^*fl/fl*^ mice exhibit reduced gene expression of bicarbonate transporters (**A)** *Slc4a2*, (**B)** *Slc4a4*, and (**D)** *Slc26a6* upon loss of CFTR from maturation-stage ameloblasts. (**C)** *Slc26a1* was also reduced, though statistically insignificant. The proton transporter (**E)** *Slc9a1* is also downregulated. The cytoplasmic localizing (**F)** *Car2* enzyme is reduced, but the secreted (**G)** *Car6* is significantly upregulated. Fold change was calculated relative to *Odam-Cre* by the ΔΔCT method, followed by the two-tailed, unpaired t-test. *p* < 0.05 is statistically significant

To assess whether this loss in ameloblast modulation and pH cycling coincides with changes to pH regulation, we evaluated the gene expression of known pH regulators expressed in maturation-stage ameloblasts. Bicarbonate likely enters ameloblasts through the basolateral membrane via the NBCe1 (*Slc4a4*) sodium/ bicarbonate symporter and released into the enamel matrix through chloride/ bicarbonate exchangers such as AE2 (*Slc4a2*) on the basolateral membrane or through SAT1 (*Slc26a1*) and Pendrin L1/ PAT1 (*Slc26a6*) on the apical, enamel-facing membrane [14, 23, 24]. When CFTR was knocked out from maturation-stage ameloblasts, we observed a transcriptional downregulation of all these bicarbonate transporters (Fig. 5A-D), though *Slc26a1* was statistically insignificant. Additionally, the basolateral sodium/ hydrogen antiporter NHE1 (*Slc9a1*) was downregulated (Fig. 5E) [14, 15]. Further assessment of the intracellular carbonic anhydrase *Car2* (Fig. 5F) found it to be downregulated upon loss of CFTR while the secreted *Car6* (Fig. 5G) was significantly upregulated [14, 20].

### Lack of Functional CFTR Disrupts Matrix Protein Hydrolysis, KLK4 Expression, and Endocytosis of Enamel Matrix Proteins During the Maturation Stage

**Fig. 6:**
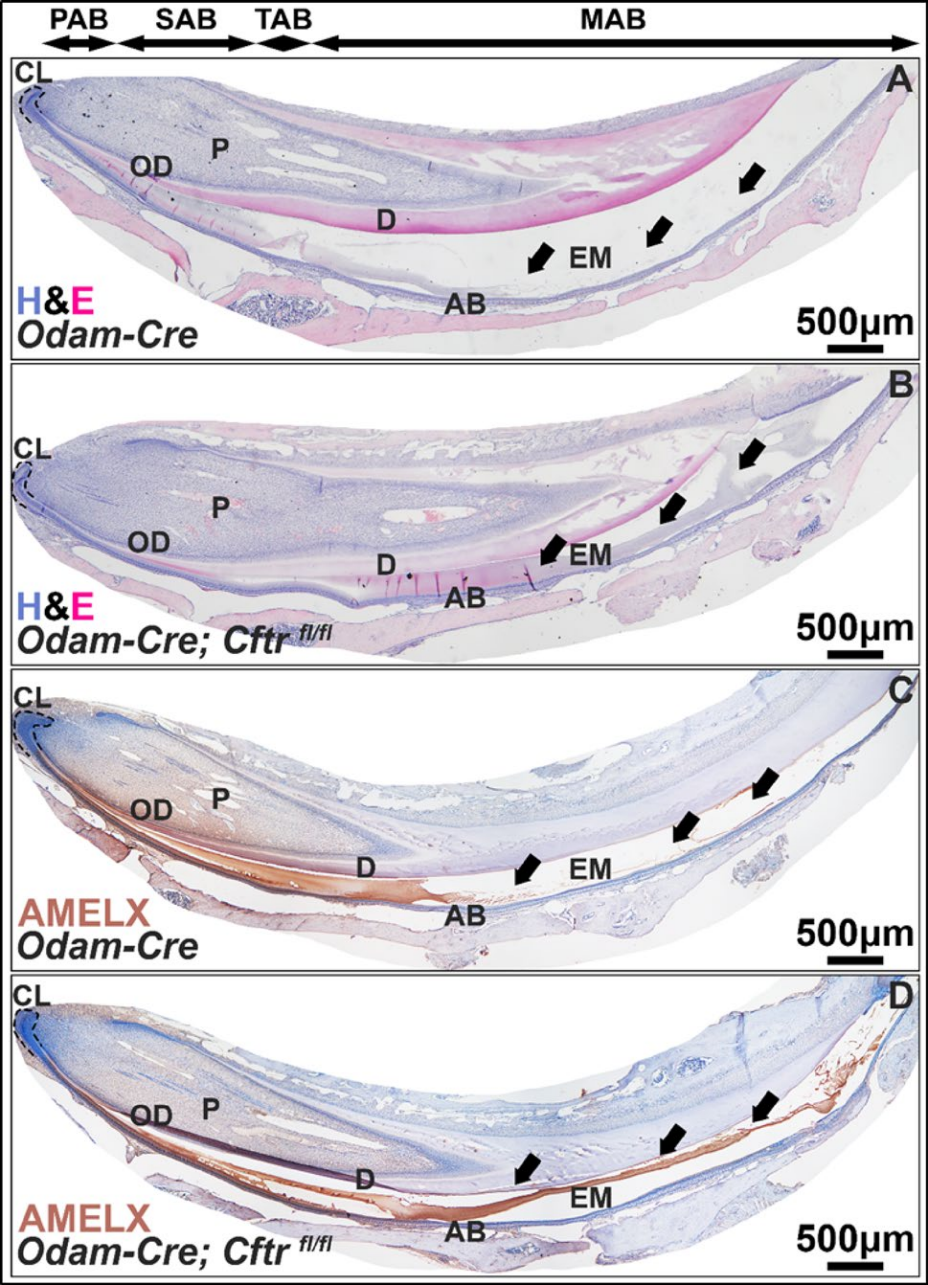
**Histological analyses of enamel matrix in CFTR-deficient enamel organs.** 4x stitches of hematoxylin and eosin (H&E) stained mouse hemimandibles sections show an absence of organic matrix components in (**A)** *Odam-Cre* controls that is retained in (**B)** the *Odam; Cre; Cftr* ^*fl/fl*^ enamel compartment (see arrows). (**C-D**) This matrix is positive (brown) for AMELX. CL, cervical loop; OD, odontoblasts; P, pulp; D, dentin; AB, ameloblasts; EM, enamel matrix

Matrix protein processing and reuptake are critical functions of maturation ameloblasts. H&E stained hemimandibles from *Odam-Cre* mice (Fig. 6A) and *Odam-Cre; Cftr* ^*fl/fl*^ mice (Fig. 6B) showed retention of enamel matrix proteins upon loss of CFTR (see arrows), which were immunopositive for amelogenin (AMELX) (Fig. 6C-D). See Supplemental Fig. 5 for immunohistochemistry negative controls.

**Fig. 7:**
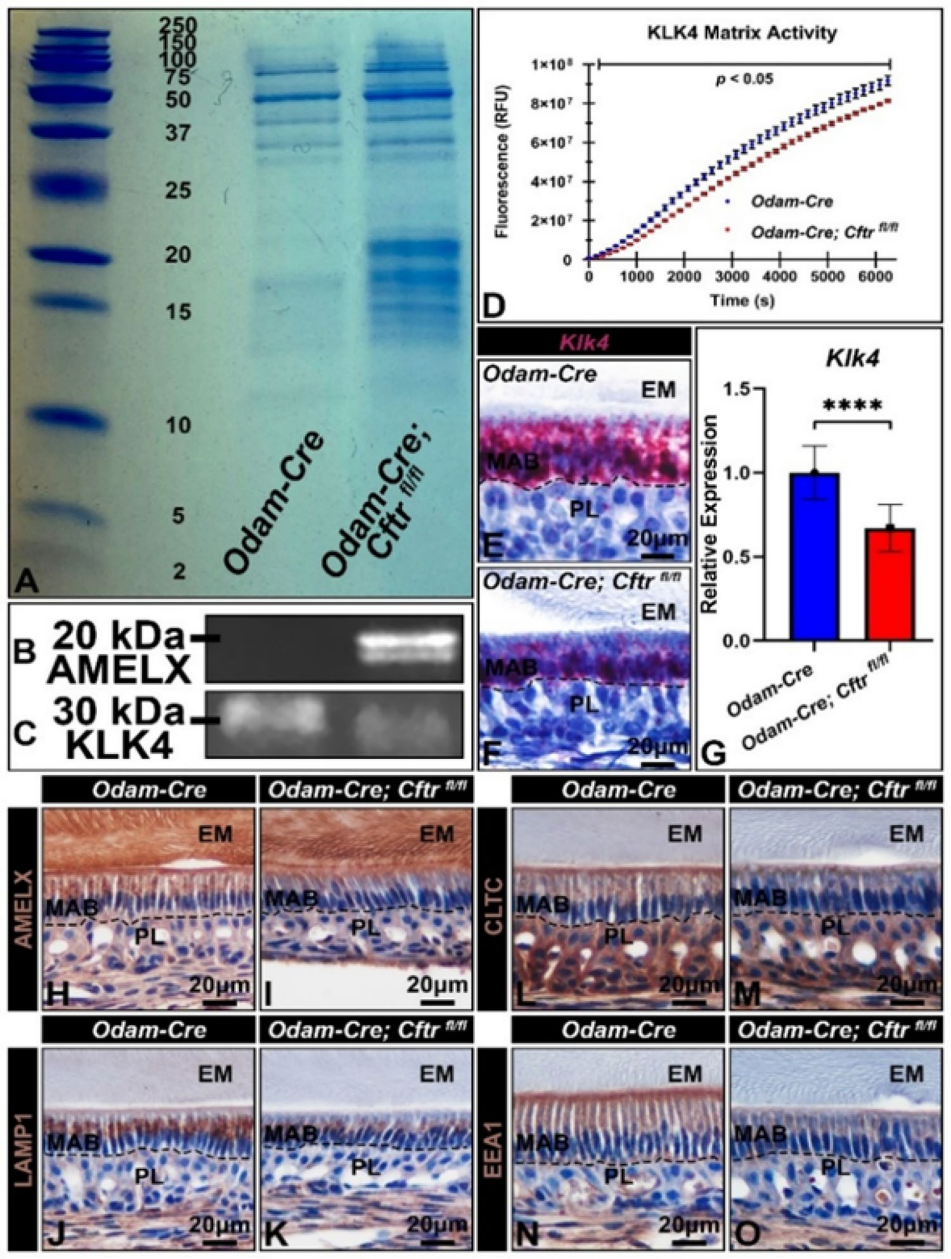
**Assessment of CFTR loss-of-function on enamel matrix protein processing/ reuptake. (A)** Representative image of enamel matrix protein extracts from microdissected *Odam-Cre* and *Odam-Cre; Cftr* ^*fl/fl*^ maturation-stage mandibular incisor fragments run on SDS-PAGE and stained with Coomassie blue. (**B)** Immunoblotting with anti-AMELX polyclonal antibody demonstrates positive signal in *Odam-Cre; Cftr* ^*fl/fl*^ extracts that is absent in controls. (**C)** Immunoblotting with anti-KLK4 antibody exhibits a reduction in KLK4 in conditional knockout matrix extracts.(**D)** Incubation of maturation-stage mandibular incisor fragments with fluorogenic quench peptide Boc-Val-Pro-Arg-AMC found that CFTR conditional knockout incisors have reduced KLK4 activity compared to controls, *p <* 0.05 is significant. (**E-F**) Representative RNAScope image of sagittal sections of control and conditional knockout mouse hemimandibles with *Klk4* probes and (**G)** reverse transcription quantitative polymerase chain reaction (RT-qPCR) revealed a reduction in *Klk4* gene expression in microdissected maturation-stage incisor enamel organs. **** *p* < 0.05. Fold change calculated by the ΔΔCT method, followed by the two-tailed, unpaired t-test. (**H-I**) 40x images of AMELX staining exhibit reduced of intracellular amelogenin in knockout ameloblasts versus controls. Immunostaining for (**J-K**) LAMP1, (**L-M**) CLTC, and (**N-O**) EEA1, revealed significant reductions in the protein abundance of mediators of the endo-lysosomal degradative pathway upon loss of CFTR

SDS-PAGE of incisor matrix protein extracts showed increased amounts of higher molecular weight amelogenins (around 20 kDa) in conditional knockout incisors relative to controls (Fig. 7A). When these extracts were subjected to western blotting, AMELX could only be detected in the conditional knockout extracts (Fig. 7B). KLK4, the major enamel matrix proteinase expressed during the maturation stage, was also reduced in the enamel matrix of CFTR-deficient enamel organs (Fig. 7C). This reduction in KLK4 corresponds with a reduction in KLK4 matrix activity, when microdissected incisor fragments were incubated ex vivo with the fluorogenic quench substrate Boc-Val-Pro-Arg-AMC, a substrate that is specifically cleaved by KLK4 and serves as a functional enzymatic readout (Fig. 7D). Concordantly, when gene expression of *Klk4* in CFTR conditional knockout ameloblasts was examined, we found a significant reduction of the proteinase by in situ hybridization RNAScope (4 replicate samples) (Fig. 7E-F). and by RT-qPCR (Fig. 7G).

In addition to matrix enzymatic cleavage, maturation-stage ameloblasts also serve to reuptake these hydrolyzed products through the endo-lysosomal degradative pathway [17, 34]. Control ameloblasts stained positive for intracellular AMELX (Fig. 7H) and is reduced with CFTR loss-of-function (Fig. 7I). Loss of CFTR function also led to a reduction of lysosome-associated membrane protein 1 (LAMP1), clathrin heavy-chain (CLTC), and early endosome antigen 1 (EEA1), all of which are markers of the endo-lysosomal degradative pathway (Fig. 7K-O).

## Discussion

Tooth enamel is the most highly mineralized tissue in vertebrates and is formed by the enamel organ, the only epithelial-derived tissue capable of biomineralization under non-diseased states. Unique to amelogenesis is the cycling of extracellular matrix pH that occurs as enamel matures, coinciding with the modulation between ruffle-ended and smooth-ended ameloblasts [11–13, 16]. While buffering of protons released from hydroxyapatite formation is thought to facilitate matrix protein hydrolysis and mineral accretion, the precise mechanisms that drive ameloblast modulation and pH cycling have yet to be fully elucidated [14, 19, 20]. Bicarbonate-mediated buffering of the extracellular matrix CFTR has been proposed to regulate enamel matrix pH [14, 21].

CFTR is a chloride and bicarbonate channel previously identified in ameloblasts [9, 10]. In humans, mutations in *CFTR* cause cystic fibrosis (CF) [4, 5]. CF-associated enamel defects have been reported since the 1960s and are attributed to either direct effects of the disease itself or consequences of therapeutic agents, such as tetracycline-related enamel discoloration and hypoplasia [35]. In conjunction with contributions of secondary effects from other organ systems, these factors underscore the complicated etiology of CF-associated enamel defects and raises questions regarding the direct role of CFTR in enamel formation [6].

Given the multisystem and extrinsic factors associated with CFTR loss-of-function, we utilized available *Odam-Cre* and *Cftr* ^*fl/fl*^ mouse lines to create conditional CFTR loss-of-function driven by the *Odam* promotor. Both CFTR and ODAM are expressed in a similar pattern in maturation-stage ameloblasts, allowing us to determine how CFTR loss of function affects enamel maturation. We crossed the *Odam-Cre* mouse line with a *Cftr* ^*fl/fl*^ mouse line, resulting in the excision of *Cftr* exon 11 and the generation of an in-frame mRNA but functionally null CFTR [30]. We found changes in maturation-stage ameloblast morphology, whereas secretory stage ameloblasts appeared to be unaffected. However, similar to reports of enamel defects in a CFTR global knockout mouse, enamel in *Odam-Cre; Cftr* ^*fl/fl*^ mice was hypomineralized and the matrix remained acidified [10].

Our analysis of maturation-stage ameloblasts revealed that this change to the enamel matrix pH was associated with the loss of ameloblast modulation, as indicated by a loss in proteins associated with the epithelial junctional complexes found in ruffle-ended ameloblasts. Junctional proteins are critical for barrier function and selective permeability of solutes into the forming enamel matrix [13, 15, 16]. Therefore, distal localization of tight and gap junctions (as indicated by CLDN1 and GJA1/ Cx43 expression) in maturation-stage ameloblasts are key markers of ruffle-ended ameloblasts and were noticeably lost as enamel maturation progressed. Since the ameloblast ruffled border is also defined by actin-rich membrane structures, the lack of beta actin staining at the apical edge of CFTR-deficient ameloblasts — in conjunction with disrupted tight/ gap junctions — suggested to us that maturation-stage ameloblasts were arresting at a smooth-ended-like morphology, which lacks apical invaginations and junctional complexes.

In other cells, CFTR has been shown to bind to and regulate the formation of cell junction complexes, as well as directly engaging with the actin cytoskeleton to maintain cellular polarity [36–38]. Therefore, in ameloblasts, CFTR may also specifically function in the formation of cell junction complexes that are required for the formation of the ruffle-ended ameloblast morphology. Consistent with the loss of ruffle ended cell morphology, we found a loss of apical membrane localization of both the V-ATPase, which is thought to play a role in both endocytosis and acidification of vesicular and extracellular compartments during enamel formation [15], and NCKX4 which is associated with calcium transport into the forming enamel matrix in *Odam-Cre; Cftr* ^*fl/fl*^ maturation-stage ameloblasts [33]. This contrasts with control ameloblasts, which localize these transporters to the apical membrane of ruffle-ended cells and then subsequently traffic/ re-localize them to the cytosol of smooth-ended cells.

An interesting finding in our study was the effect of CFTR loss of function to reduce KLK4 synthesis. CFTR is known to regulate FOX01, a protein important in the regulation of energy metabolism [39, 40]. FOX01 has also been shown to regulate *Klk4* synthesis in vitro [41], suggesting the possibility that reduction in *Klk4* expression in CFTR loss-of-function ameloblasts may be directly related to CFTR mediated gene expression. Future studies will be needed to functionally assess the relationship between FOX01, CFTR, and KLK4. KLK4 activity, which has previously been shown to increase in an acidic pH [33], was reduced in CFTR loss-of-function, contributing to the retention of higher molecular weight amelogenin in the enamel matrix of mouse incisors. In addition, the reduction in endocytosis related proteins that are associated with maturation-stage ameloblasts indicates that in CFTR loss-of-function, endocytosis of enamel matrix proteins is also reduced.

In incisors, gene expression of the bicarbonate importer *Slc4a4*, chloride/ bicarbonate exchangers *Slc4a2* and *Slc26a6*, sodium/ proton antiporter *Slc9a1*, and the bicarbonate-generating *Car2* were all significantly downregulated. Our observations are reflective of transcriptional changes also observed in CFTR-ablated intestinal organoids (enteroids) and is consistent with a compensatory response to increased intracellular chloride and bicarbonate concentrations, resulting from a lack of anion conductance by CFTR into the enamel matrix [42]. This is further supported by electron probe microanalysis of CFTR global knockout enamel and electrophysiological studies demonstrating increased intracellular chloride concentrations, a more alkaline intracellular environment, and reduced chloride conductance in CFTR-deficient tissues [10, 30, 42]. These transcriptional changes suggest that the ameloblast transepithelial network of chloride and bicarbonate transport is disrupted and ascribes a potential role of CFTR in maintaining intracellular homoeostasis of these anions, in addition to regulating extracellular enamel matrix pH.

Interestingly, we also found that mouse molars, while still hypomineralized based on qualitative assessment, were much less affected by conditional ablation of CFTR, a phenomenon that has also been observed by previous studies on CFTR global knockout mice [8, 10]. This phenotypic dimorphism may highlight inherent differences between molar and incisor development. Unlike molars, incisors are continuously erupting, and as such have increased biomechanical stress as the tooth moves through the alveolar bone, which may differentially affect gene expression in the enamel organ [43]. These effects may be compounded by the increase in inflammation associated with eruption [44] and enhance the increase in cellular stress known to be associated with CFTR loss-of-function [45]. Understanding the differences in the incisor and molar mouse models are important in assessing questions related to loss-of-function mouse models, and future studies comparing molar and incisor gene expression, such as in the CFTR loss-of-function mouse model, may help to further understand the differences between these model systems.

In summary, our findings show multiple roles of CFTR in enamel maturation. CFTR appears to be critical in regulating ameloblast modulation, which entails the formation and localization of junctional complexes and ion transporters to properly form the ameloblast ruffled border. With CFTR loss-of-function, ameloblasts arrest in the more quiescent smooth-ended morphology, which is associated with reductions in endocytosis and ion transport. In addition, CFTR may have an indirect role in matrix hydrolysis, potentially through regulating transcription of *Klk4*. While CFTR immunolocalizes to the distal end of both ruffle-ended and smooth-ended ameloblasts, and further studies are needed to compare its activation in ruffle ended and smooth ended ameloblasts, to assess the role of CFTR in ameloblast modulation.

## Supplementary Information

Below is the link to the electronic supplementary material.

Supplementary Material 1
