# Supplementary material for: The Role of the Cystic Fibrosis Transmembrane Conductance Regulator (CFTR) in Enamel Formation
Source: Calcif Tissue Int. 2026 May 4;117(1):76. doi: 10.1007/s00223-026-01539-1 (PMC13139227; doi:10.1007/s00223-026-01539-1)
Supplement: Supplementary file 1 — Supplementary Material 1 [file 223_2026_1539_MOESM1_ESM.docx]

| **Supplemental Table 1. PCR Primer Sequences** | | |
| --- | --- | --- |
| **Target** | **Amplicon Size** | **Sequence (5’🡪 3’)** |
| **Genotyping PCR Primers** | | |
| **C57BL/6J-*Odam^em1(cre)Mlp^*/Mmjax** | ***Wt:* 330 bp**  ***Mut: ~*200 bp** | **Common FOR: GCCTCAACTTGGATAAAGCAG** |
|  |  | ***Wt* REV: GCATGGTGTTTGTAGGCAGAT** |
|  |  | ***Mut* REV: ACACCGGCCTTATTCCAAG** |
| ***Cftr^tm1Cwr^*** | ***Wt:* 361 bp**  ***Fl11:* 408 bp**  **Δ11: 154 bp** | **P1: GTAGGGGCTCGCTCTTCTTT** |
|  |  | **P2: GTACCCGGCATAATCCAAGA** |
|  |  | **P3: AGCCCCTCGAGGGACCTAAT** |
| **RT-PCR Primers** | | |
| ***mCftr* Exons 9-12** | ***Wt:* 563 bp**  **Δ11: 371 bp** | **FOR: CCACAGGCATAATCATGGAA** |
|  |  | **REV: TGTGACTCCACCTTCTCCAA** |
| **RT-qPCR Primers** | | |
| ***mCar2* (CAII)** | **139 bp** | **FOR: TGCGGCCTTTGCTAACTTC** |
|  |  | **REV: GCTGACAGTAATGGGCTCCC** |
| ***mCar6* (CAVI)** | **163 bp** | **FOR: CGGGACCTATGAGAATGCC** |
|  |  | **REV: GGTCGTGTCTTTCAGAGTTG** |
| ***mCftr* (CFTR)** | **144 bp** | **FOR: GTCCAGCCTGTCTTGCTAGG** |
|  |  | **REV: GCCAAAAATAGCTGGGTGAA** |
| ***mKlk4* (KLK4)** | **102 bp** | **FOR: CGGGAGTCTTGGTGCATCC** |
|  |  | **REV: CTTGGGAGCCTTTCAGGTTATG** |
| ***mOdam* (ODAM)** | **189 bp** | **FOR: TAGCCCTATGTCCTATGTGG** |
|  |  | **REV: GGTGGTGCGAATCCAAATTG** |
| ***mSlc4a2* (AE2)** | **111 bp** | **FOR: GACTTCCGCGATGCACTTG** |
|  |  | **REV: CTTTGTCTTCTCCCCCAGTA** |
| ***mSlc4a4* (NBCe1)** | **129 bp** | **FOR: GGTCACCACACGATCTACATTG** |
|  |  | **REV: TTTGTCGGAGTAGTTCTCGGA** |
| ***mSlc9a1* (NHE1)** | **123 bp** | **FOR: GATTTACCTCTCACATCCGG** |
|  |  | **REV: CTACTCCTGAGGCGATGAG** |
| ***mSlc26a1* (SAT1)** | **137 bp** | **FOR: GTTGCCACTGCCCTTACTC** |
|  |  | **REV: GTCAAGATGGTCACAGAAGC** |
| ***mSlc26a6* (Pendrin L1)** | **158 bp** | **FOR: GGAACTACTCACGCTCATTG** |
|  |  | **REV: CAAAGGCATTTCCCACAAGC** |


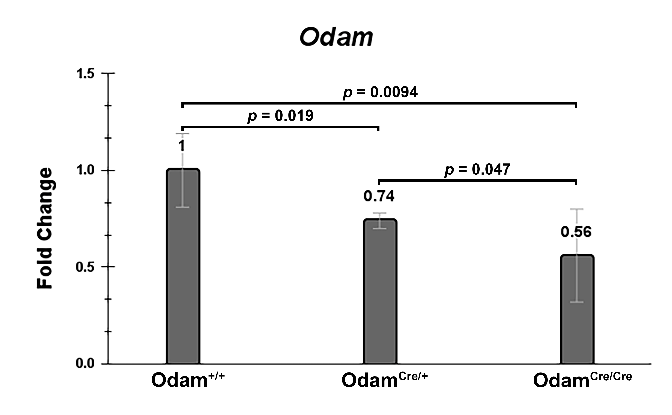


**Supplemental Fig. 1 *Odam* expression in P10 *Odam-Cre* first molar enamel organs.** RT-qPCR analysis of *Odam* expression in *Odam-Cre* mice, either hemizygous or homozygous for the *Odam-Cre* allele. Fold change was calculated relative to *Odam^+/+^* by the ΔΔCT method, followed by the two-tailed, unpaired t-test, with Bonferroni correction following ANOVA. *p <* 0.016 is significant. Standard deviation is based on ΔCT values.

| **Supplemental Table 2. Antibodies** | | | | |
| --- | --- | --- | --- | --- |
| **Target** | **Vendor** | **Catalog #** | **Application** | **Dilution Factor** |
| **ACTB** | **Invitrogen** | **MA1-91399** | **IHC** | **1:1000** |
| **AMELX** | **Den Besten Lab** | **N/A** | **IHC**  **WB** | **1:250**  **1:500** |
| **ATP6V1A** | **Abcam** | **ab199325** | **IHC** | **1:500** |
| **CFTR** | **Invitrogen** | **MA5-11768** | **IHC** | **1:50** |
| **CLDN1** | **Abcam** | **ab13098** | **IHC** | **1:450** |
| **CLTC** | **Abcam** | **ab21679** | **IHC** | **1:200** |
| **EEA1** | **Proteintech** | **28347-1-AP** | **IHC** | **1:300** |
| **GJA1/ Cx43** | **Novus** | **NBP2-38234** | **IHC** | **1:150** |
| **KLK4** | **Abcam** | **ab231048** | **WB** | **1:500** |
| **LAMP1** | **Abcam** | **ab24170** | **IHC** | **1:750** |
| **NCKX4** | **NeuroMab** | **75-404** | **IHC** | **1:500** |
| **ODAM** | **Proteintech** | **16508-1-AP** | **IHC** | **1:400** |

**
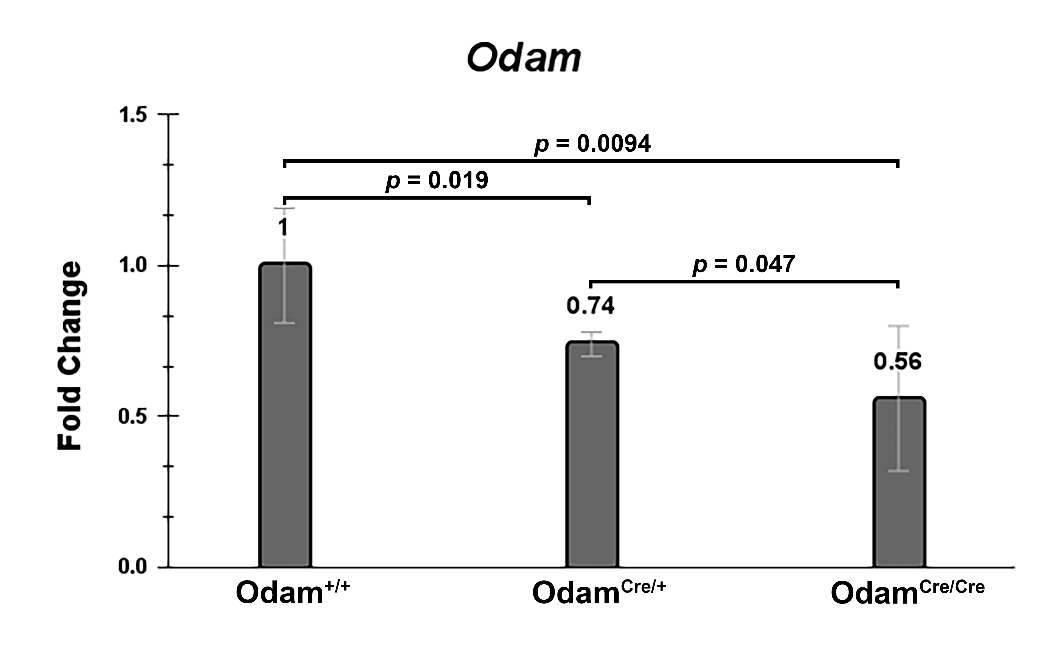

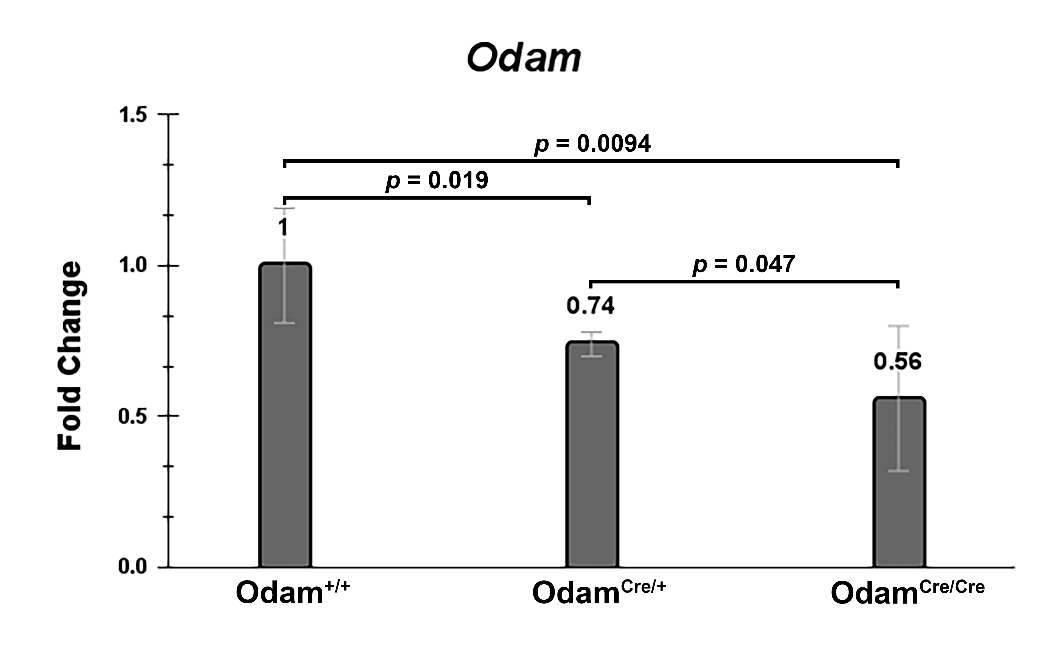
**


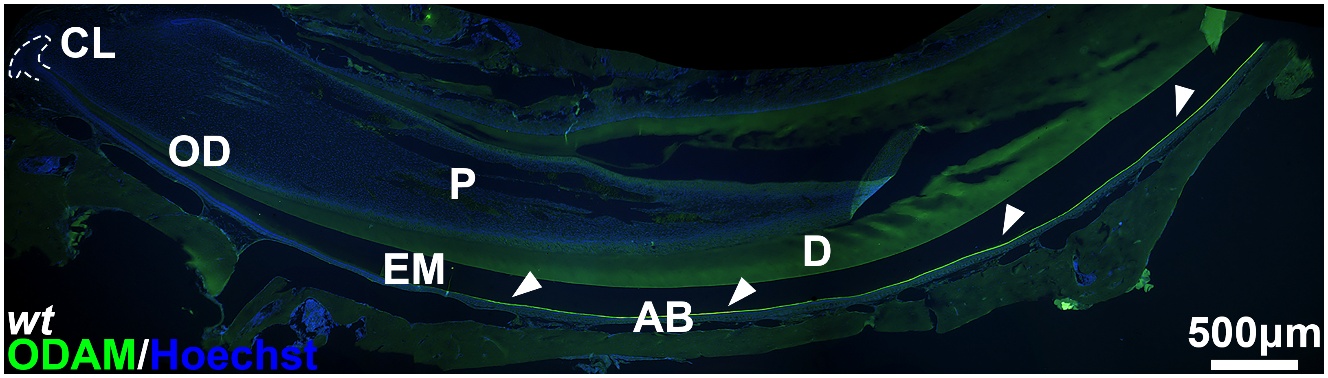


**Supplemental Fig. 2 Localization of ODAM in *wt* mouse incisor enamel organs.** Representative 4x stitch of *wt* mouse hemimandibles stained with polyclonal rabbit anti-ODAM antibody. ODAM staining is in green and nuclei are in blue. ODAM begins to be detected during the secretory to maturation transition and is detected throughout the maturation stage, localizing specifically to the distal-end of maturation-stage ameloblasts. AB, ameloblasts; CL, cervical loop; D, dentin; EM, enamel matrix; OD, odontoblasts; P, pulp.

**Supplementary Fig. 1 Expression analysis of *Odam* in *Odam-Cre* enamel organs.**

**Supplementary Fig. 1 Expression analysis of *Odam* in *Odam-Cre* enamel organs.**


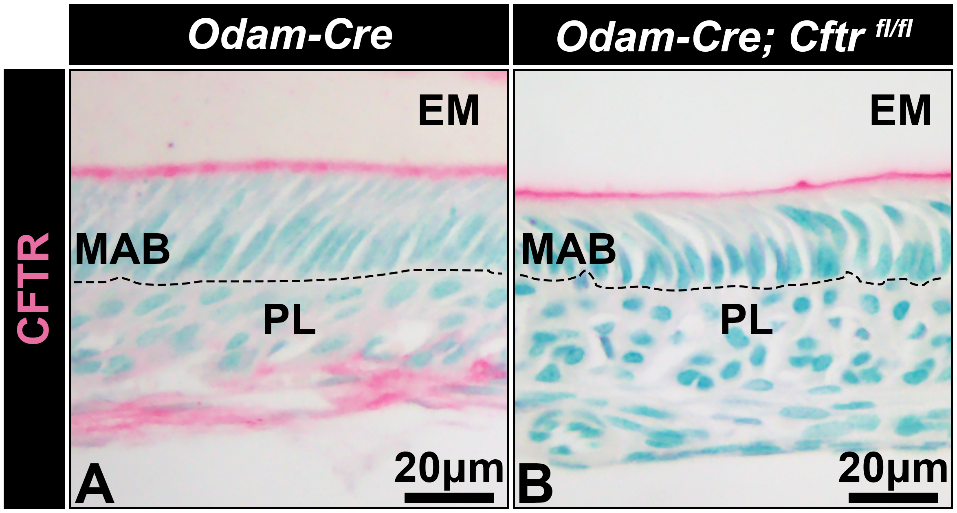


**Supplemental Fig. 3 Characterization of CFTR expression in *Odam-Cre* and *Odam-Cre; Cftr ^fl/fl^* maturation-stage enamel organs.** Immunostaining of mandibular maturation-stage ameloblasts with mouse anti-CFTR monoclonal antibody in **(A)** *Odam-Cre* and **(B)** *Odam-Cre; Cftr ^fl/fl^*. CFTR staining was visualized in red and nuclei are in green. MAB, maturation-stage ameloblasts; EM, enamel matrix; PL, papillary layer.


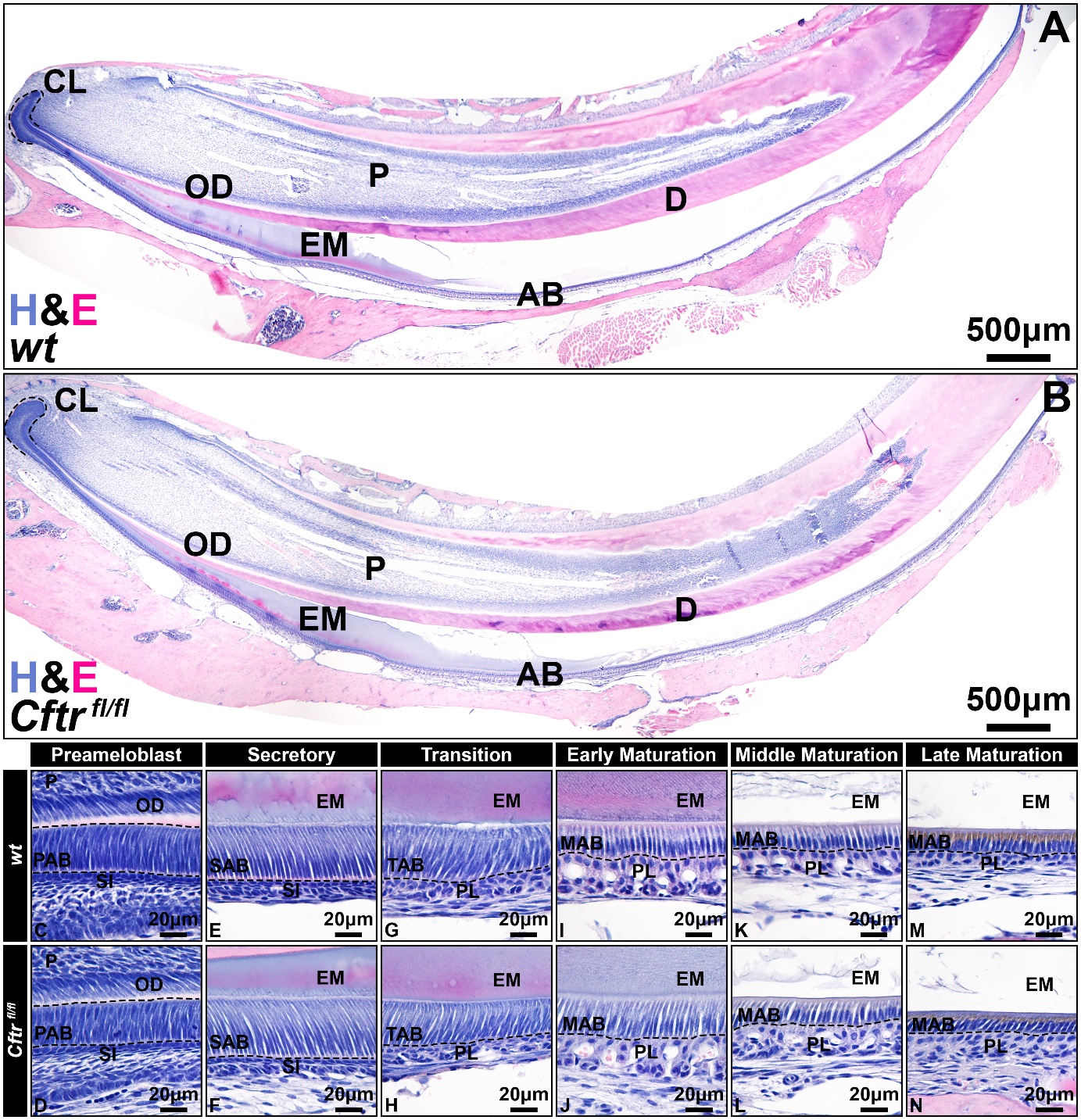


**Supplemental Fig. 4 Histological assessment of *wt* and *Cftr ^fl/fl^* incisor enamel organs.** 4x representative stitches of **(A)** *wt* and **(B)** *Cftr ^fl/fl^* hemimandibles sectioned along the sagittal plane and stained with hematoxylin (blue) and eosin (pink). AB, ameloblasts; CL, cervical loop; D, dentin; EM, enamel matrix; OD, odontoblasts; P, pulp. 40x representative images of ameloblasts at **(C-D)** preameloblast, **(E-F)** secretory, **(G-H)** transition, **(I-J)** early maturation, **(K-L)** middle maturation, and **(M-N)** late maturation stages. PAB, preameloblasts; SI, stratum intermedium; P, pulp; OD, odontoblasts; EM, enamel matrix; SAB, secretory-stage ameloblasts; TAB, transition-stage ameloblasts; MAB, maturation-stage ameloblasts.


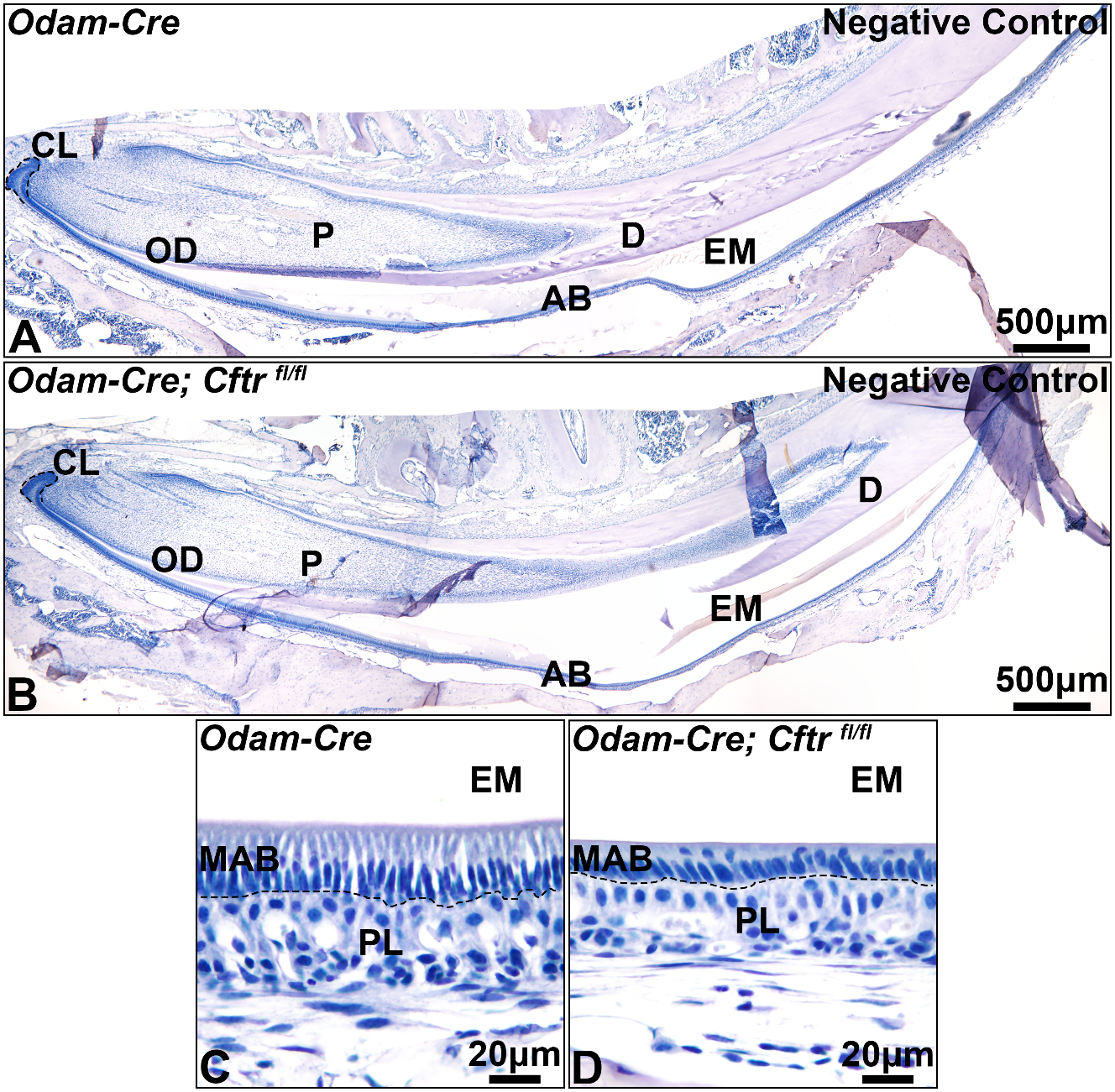


**Supplemental Fig. 5 Immunohistochemistry negative controls.** Representative 4x stitches of **(A)** *Odam-Cre* and **(B)** *Odam-Cre; Cftr ^fl/fl^* negative controls. Sagittal sections of mouse hemimandibles were incubated without primary antibody, followed by incubation with anti-rabbit HRP-conjugate secondary antibody and counterstained with hematoxylin. AB, ameloblasts; CL, cervical loop; D, dentin; EM, enamel matrix; OD, odontoblasts; P, pulp. Representative 40x images of **(C)** *Odam-Cre* and **(D)** *Odam-Cre; Cftr ^fl/fl^* maturation-stage enamel organs. MAB, maturation-stage ameloblasts; EM, enamel matrix; PL, papillary layer.
